# Non-Perturbative Identification and Subtyping of Amyloidosis in Human Kidney Tissue with Raman Spectroscopy and Machine Learning

**DOI:** 10.3390/bios13040466

**Published:** 2023-04-08

**Authors:** Jeong Hee Kim, Chi Zhang, Christopher John Sperati, Serena M. Bagnasco, Ishan Barman

**Affiliations:** 1 Department of Mechanical Engineering, Johns Hopkins University, Baltimore, MD 21218, USA; 2 Division of Nephrology, School of Medicine, Johns Hopkins University, Baltimore, MD 21287, USA; 3 Department of Pathology, School of Medicine, Johns Hopkins University, Baltimore, MD 21218, USA; 4 The Russell H. Morgan Department of Radiology and Radiological Science, School of Medicine, Johns Hopkins University, Baltimore, MD 21218, USA; 5 Department of Oncology, School of Medicine, Johns Hopkins University, Baltimore, MD 21218, USA

**Keywords:** Raman spectroscopy, machine learning, renal amyloidosis, human kidney tissue, amyloid subtyping

## Abstract

Amyloids are proteins with characteristic beta-sheet secondary structures that display fibrillary ultrastructural configurations. They can result in pathologic lesions when deposited in human organs. Various types of amyloid protein can be routinely identified in human tissue specimens by special stains, immunolabeling, and electron microscopy, and, for certain forms of amyloidosis, mass spectrometry is required. In this study, we applied Raman spectroscopy to identify immunoglobulin light chain and amyloid A amyloidosis in human renal tissue biopsies and compared the results with a normal kidney biopsy as a control case. Raman spectra of amyloid fibrils within unstained, frozen, human kidney tissue demonstrated changes in conformation of protein secondary structures. By using t-distributed stochastic neighbor embedding (t-SNE) and density-based spatial clustering of applications with noise (DBSCAN), Raman spectroscopic data were accurately classified with respect to each amyloid type and deposition site. To the best of our knowledge, this is the first time Raman spectroscopy has been used for amyloid characterization of ex vivo human kidney tissue samples. Our approach, using Raman spectroscopy with machine learning algorithms, shows the potential for the identification of amyloid in pathologic lesions.

## 1. Introduction

Amyloidosis is an uncommon systemic disease caused by irregular protein aggregation and misfolding that leads to the formation of insoluble amyloid deposits [1,2,3,4]. Different types of amyloid derive from various amyloid precursor proteins and can infiltrate various organs [1,5]. Although these protein deposits and their sequences vary, amyloid fibrils share a common structure, namely steric zippers, arranged in a periodic fibrillar lattice of β-sheets; this structure can be observed across various modalities, including NMR spectroscopy, cryo-electron microscopy (cryo-EM), and atomic force microscopy (AFM) [6,7,8]. 

Recently, Raman spectroscopy has been utilized to study amyloid fibril formation and structural conformations [9,10,11,12,13]. By vibrationally fingerprinting biological samples at a molecular level, Raman spectroscopy identifies various molecules, including proteins and lipids, with high sensitivity and in a nondestructive and label-free manner [14,15,16,17,18,19,20]. In addition, its relatively simple setup and the lack of a requirement for a priori knowledge of sample composition make Raman spectroscopy a potential tool to study amyloidosis. Previous studies have shown that Raman spectroscopy is sensitive to differences in structural conformations of different amyloid types [11,12,21,22,23]. In particular, amide I and III bands identified β-sheet structures in both amyloid fibrils isolated from patients and synthesized amyloid peptides [9,10,22,24]. However, although these findings established the applicability of Raman spectroscopy to study amyloidosis, synthesized amyloid and isolated amyloid fibrils are overly simplified and disconnected from protocols of clinical detection and diagnosis.

To address this limitation, several researchers have investigated amyloid deposits in tissue with Raman spectroscopy. Animal models have been used to identify biomarkers representative of the amyloid signature within a mixture of biomolecules, coupled with spectral unmixing analysis [25,26,27]. In addition, others have applied Raman spectroscopy to tissue biopsies of patients that reported changes in the protein signature associated with amyloid [24,28,29,30,31,32,33]. Although these studies demonstrate Raman spectroscopy’s capability to distinguish subtle spectral changes due to amyloid deposits in tissue samples, they were mainly focused on brain tissues to investigate amyloid involvement with disorders such as Alzheimer’s disease and Parkinson’s disease. However, no previous Raman spectroscopic investigations of renal amyloid deposits exist, despite the fact that the kidney is one of the most commonly involved organs in amyloidoses [5,34].

Here, we employ Raman spectroscopy to examine amyloid deposits for the first time, to the best of our knowledge, in unstained fresh-frozen human kidney tissues. Specifically, we investigated immunoglobulin light chain (AL) and serum amyloid A (AA), which are precursor proteins that give rise to AL amyloidosis and AA amyloidosis, respectively [3,35]. These amyloid diseases represent the two major amyloid diseases with kidney involvement [5,36,37]. We investigated the Raman spectra of AL, AA, and non-amyloidogenic (NA) tissues collected from six patients through analyses of the protein band area and second derivative. Then, using t-distributed stochastic neighbor embedding (t-SNE) and density-based spatial clustering of applications with noise (DBSCAN), we characterized endogenous molecular compositions and structures indicative of amyloid deposits and demonstrated heterogeneity between different amyloid types. In this study, we describe in detail our methodological approach, combining Raman spectroscopy with machine learning techniques to identify and characterize the two major types of amyloidosis in human renal tissue.

## 2. Materials and Methods

### 2.1. Sample Preparation

**Figure 1:**
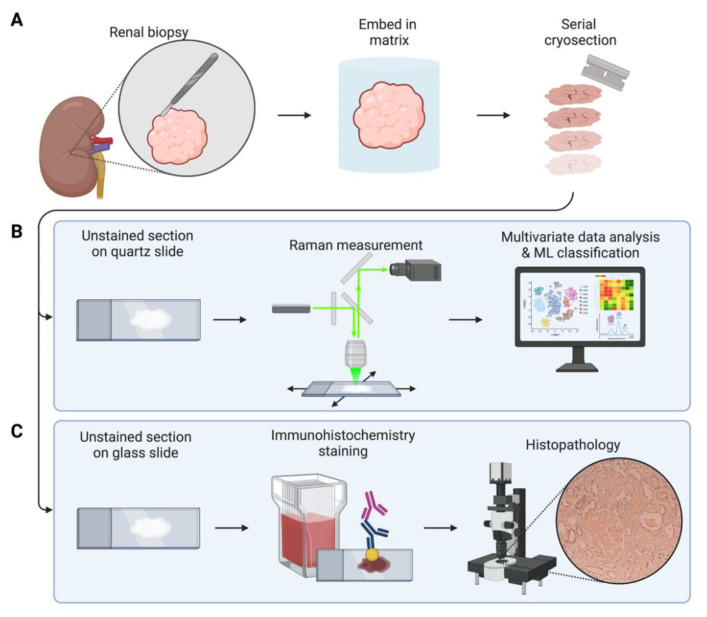
The overall workflow for amyloid identification and subtyping. (**A**) Tissue preparation steps. Biopsied tissues were frozen and sectioned for evaluation. (**B**) Raman spectroscopic data acquisition and analysis. Fresh frozen tissue sections were prepared on quartz slides to minimize spectral interference, and employed for Raman measurements, which were subjected to machine learning analysis. (**C**) Histopathologic validation. Consecutive tissue sections used in (**B**) were utilized for the gold standard, immunohistochemistry evaluation. (Created with BioRender.com (accessed on 23 February 2023)).

**Figure 2:**
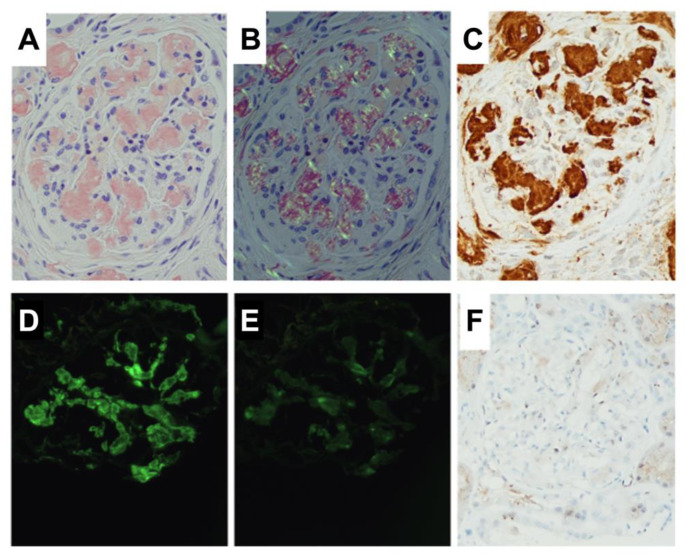
Renal amyloidosis. (**A**) Glomerular and arteriolar deposits of amyloid identified as Congo red-positive material (magnification 400×). (**B**) Glomerular amyloid Congo red-positive deposits showing birefringence under polarized light (magnification 400×). (**C**) AA amyloidosis: the immunohistochemical stain for amyloid A is strongly positive in the glomerulus and in the arterioles (magnification 400×). (**D**) AL amyloidosis: by immunofluorescence, a glomerulus containing Congo red-positive material (not shown) shows a positive stain for the kappa light chain (magnification 400×). (**E**) The immunofluorescence stain for lambda light chain is negative in the same glomerulus (magnification 400×). (**F**) The immunohistochemical stain for amyloid A is negative in the glomeruli containing deposits of AL amyloid.

Remnant, de-identified tissues from kidney biopsies performed for diagnostic purposes (IRB approval: IRB00090103) were used for this study, as illustrated in Figure 1. The biopsied tissues of AA, AL, and NA amyloidosis from 6 patients were prepared as a frozen tissue block. Fresh frozen blocks were sectioned by a cryostat, and thin-sliced kidney tissue sections were placed on quartz and glass microscope slides for Raman measurements and histological evaluation, respectively. Tissue sections for Raman measurements remained unstained and were prepared on quartz slides to avoid spectral interference with the biochemical fingerprints of the tissue sample. Consecutive slices from each tissue block were used to detect and identify amyloid fibrils through histological evaluation (Figure 2).

### 2.2. Raman Spectroscopy

A Raman spectroscopy system (Horiba Jobin Yvon-XploRA PLUS) collected Raman spectra of ex vivo kidney tissue samples (Figure 1b). A 532 nm laser was projected onto room-temperature kidney sections, and the resulting Raman scattering between 700 and 3500 cm^−1^ was recorded through a CCD camera. Measurements were taken at various pathological sites, including glomeruli and other structures within the cortical region.

The collected Raman spectra were processed using MATLAB 2018b (MathWorks, Inc., Natick, MA, USA) with baseline and background correction [38], spectral smoothing through a Savitzky-Golay filter [39], and normalization based on water content (3100–3400 cm^−1^). For multivariate and machine learning analysis, the biological fingerprint region (800–1800 cm^−1^) was selected, which contains molecular information including proteins, lipids, and other tissue constituents.

### 2.3. Data Analysis

The collected Raman spectroscopic signals were examined to identify spectral features unique to a particular amyloid type.

Second derivative analysis, which has been used to estimate the contribution of protein secondary structure [29,40], was applied to identify spectral features arising from amyloid fibrils within tissues. Second derivative spectra were obtained by the Savitzky-Golay filter [39], followed by robust locally weighted smoothing.

To further characterize spectral features associated with AL and AA amyloidosis beyond those apparent upon visual inspection, we employed t-Distributed Stochastic Neighbor Embedding (t-SNE), a multivariate analysis technique, and density-based spatial clustering of applications with noise (DBSCAN), an unsupervised machine learning approach. These allowed the unveiling and decomposing of subtle and complex tissue information with greater sensitivity by addressing spectral interference due to background and fluorescence. Both approaches considered Raman spectra collected from both glomerular and non-glomerular regions in AL, AA, and NA tissues. All analyses were performed and visualized using MATLAB and Orange [41].

Briefly, t-SNE is a dimensionality reduction technique that evaluates complicated Raman spectra. By extracting both linear and non-linear features from Raman spectra, it reduces tissue spectra containing information about various biological molecules, from a higher to a lower dimension [42]. We used a perplexity of 15 and an exaggeration of 2 as parameters.

DBSCAN is an unsupervised machine learning approach for data clustering. This machine learning technique is robust to outliers, which makes it a suitable approach for analyzing a large collection of Raman spectra. Core point neighbors and neighborhood distance (Euclidian distance) were determined based on an analysis design from a previous study [43].

## 3. Results and Discussion

To characterize amyloid deposits, we utilized Raman spectroscopy to collect molecular fingerprints of ex vivo amyloid-infiltrated human kidney tissue samples from patients affected by AL or AA amyloidosis. Raman spectra were measured both within glomeruli with amyloid deposits, which were identified by pathologists, and non-glomerular regions of tissue sections. Raman spectra of normal tissue samples (NA) were also collected as control cases for comparison. Adjacent sections of each type underwent histopathologic evaluation. Figure 1 illustrates the workflow of this study. 

### 3.1. Amide I and Amide III Bands Reveal Protein Secondary Structures Associated with Amyloidosis

**Figure 3:**
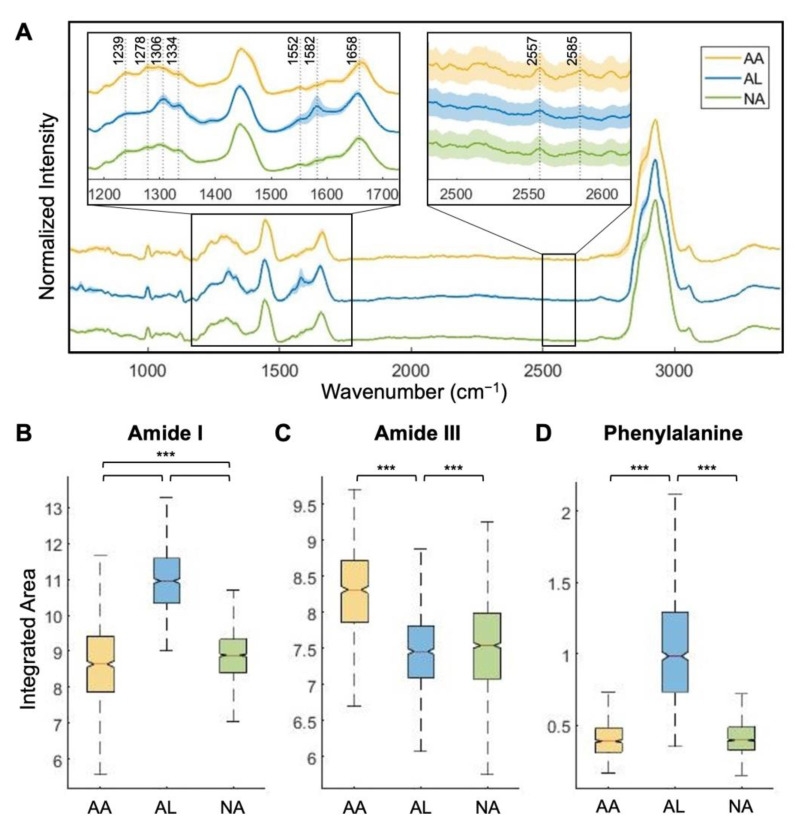
Raman spectroscopy of frozen kidney tissue featuring amyloid deposits. (**A**) Raman spectra of glomeruli within AA, AL, and NA tissues. Each spectrum represents an averaged and normalized spectrum with 1 standard deviation shaded. They are normalized on the spectral region assigned to water (3100–3400 cm^−1^), assuming an equivalent water content for all samples. Raman band area analyses of (**B**) amide I (1600–1700 cm^−1^), (**C**) amide III (1200–1300 cm^−1^), and (**D**) phenylalanine (1582 ± 3 cm^−1^) of AA, AL, and NA glomeruli. Statistical significance: *** *p* < 0.0001.

To investigate features of amyloid fibrils, Raman spectra of glomeruli within kidney tissues were obtained (Figure 3). Particularly, we observed peaks within amide I (1600–1700 cm^−1^) and amide III (1200–1300 cm^−1^) bands of protein, which are closely related to peptide backbone conformations, the main determinant of protein stability [11,21]. At amide I region, we observed a peak at 1658 cm^−1^ with AA slightly shifted to a higher (1664 cm^−1^) frequency while AL slightly shifted to a lower (1653 cm^−1^) frequency, compared to the control case. In amide III spectral region, marked changes in peaks at 1239 and 1278 cm^−1^ were observed, as peaks in AA became more distinguished whereas those in AL appeared more obscure than the NA tissue signal. Such differences are associated with secondary protein structures, particularly β-sheet and α-helix structures, which constitute amyloid fibrils [10,21,29]. The AL spectrum exhibits peaks at 1306 and 1334 cm^−1^, attributed to sidechain vibrations [11]. In addition, we observed subtle peaks in a higher wavenumber region, associated with changes in lipids. Peaks around 1552 and 1582 cm^−1^ represent aromatic amino acids, such as tryptophan and phenylalanine [21]. The intensities in the observed bands, 1582 cm^−1^ of AL tissue, and 1658 cm^−1^ of AA tissue, vary due to the non-uniform distribution of the amyloid deposits, as marked by the heterogeneity of amyloid-positive samples. In addition, the polymorphism of fibrils may augment the heterogeneity [5]. To assess the changes in protein structures arising from amyloid fibrils, Raman band areas of amide I, amide III, and phenylalanine were evaluated (Figure 3b–d). The amide I band area of AL (Figure 3b) appeared evidently higher than the others, whereas the amide III band area of AA (Figure 3c) showed a clear distinction from the others. In addition, an increase in phenylalanine band area is observed in the AL spectra (Figure 3d), with a statistically significant difference from the band area under the AA or NA tissue spectra. Such an observation indicated that both AA and AL fibrils consist of protein secondary structures with varying contributions of C-N stretching, N-H bending, and C=O stretching vibrations [21].

**Figure 4:**
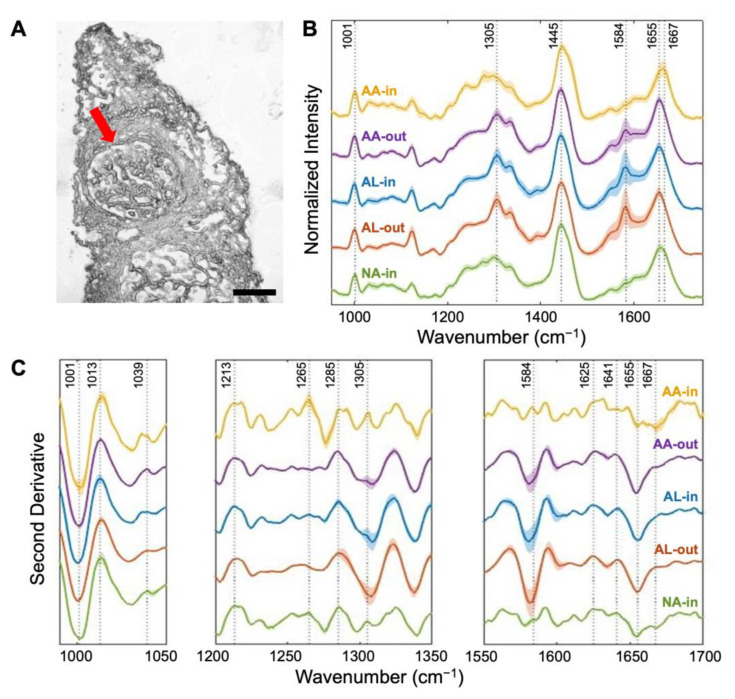
(**A**) Microscopic image of frozen kidney tissue identified with the glomerulus. Scale bar = 100 µm. (**B**) Averaged and normalized Raman spectra collected within and without glomeruli of AA, AL, and NA tissues with 1 standard deviation shaded. (**C**) Second derivative analysis of phenylalanine (1000–1500 cm^−1^), amide III (1200–1350 cm^−1^), and amide II-I (1550–1700 cm^−1^). Each spectrum in (**B**,**C**) is color-coded based on the type and deposition site and plotted in order, from top to bottom: AA-within glomeruli, AA-without glomeruli, AL-within glomeruli, AL-without glomeruli, and NA.

To further investigate the influence of amyloid fibrils depending on the associated tissue site, we expanded the examination of the Raman spectra of glomeruli, marked in Figure 4a, as well as outside of the glomerulus region. Figure 4b shows distinct spectral profiles for each amyloid type at both glomerular and non-glomerular sites. The corresponding second derivative analysis is shown in Figure 4c. We performed second derivative analysis to objectively identify sharp changes in spectra and locate their vibrational bands, enabling us to further distinguish characteristic spectral features [9,11,44]. Second derivative analysis of amide I, II, and III bands revealed spectral components and peak shifts unnoticed in Raman tissue spectra. Analysis of AA glomerular regions exhibited a split in the 1213 cm^−1^ band, with prominent peaks around 1265, 1305, and 1584 cm^−1^, associated with the mixture of β-sheet and α-helix structures. The contributions of protein secondary structures in AL fibrils were different from those in AA fibrils, with peaks observed around higher Raman bands, at 1625, 1641, and 1655 cm^−1^, mainly attributed to C=O stretching vibration. These observations are consistent with previous reports that indicate both AA amyloidosis and AL amyloidosis exhibit protein secondary structures, as the misfolded AA and AL proteins, respectively, aggregate, form amyloid fibrils, and adopt a β-sheet conformation [45]. Second derivative analyses reveal that Raman spectroscopy can molecularly distinguish this common structural feature (β-sheet) across AA and AL amyloidosis, as shown by their distinct Raman bands.

### 3.2. Machine Learning-Based Raman Spectral Analysis Can Classify Renal Amyloidosis with Respect to Deposition Sites and Types

**Figure 5:**
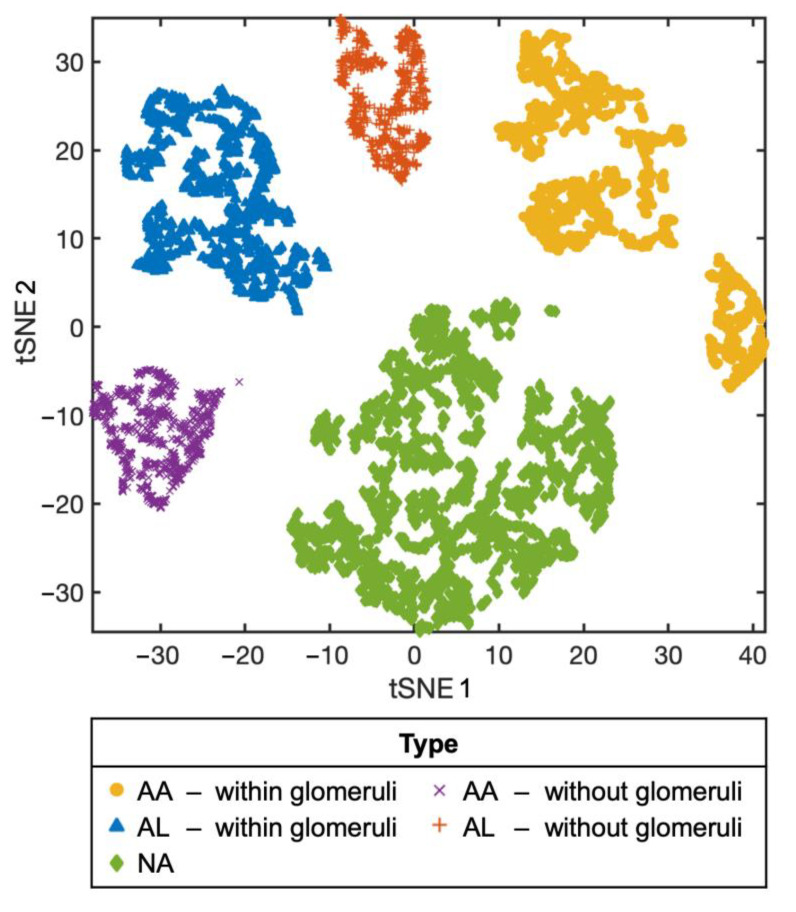
t-SNE map for the distribution of Raman spectra. Spectra were identified with their amyloid types (AA, AL, or NA) and location (within or without glomeruli). Each point represents a Raman spectrum that is positioned based on the similarity probability of the spectra in the dataset. Each group is well separated from other groups, indicating that the Raman spectra of the same group are similar and distinct from those of other groups.

To distinguish subtle intrinsic spectral differences between amyloid types that were not detected by visual inspection of the tissue spectra, we utilized a multivariate dimension reduction and data exploration technique, t-SNE. Figure 5 shows the t-SNE distribution results of the processed Raman tissue spectra of the biological fingerprint region, ranging between 800 and 1800 cm^−1^. We subjected a collection of Raman spectra to non-linear dimensionality reduction and projected them onto a lower dimension, specifically, 2-dimensional space (t-SNE components 1 and 2). The t-SNE map reveals that spectra collected from each amyloid type are clearly separated, as are spectra from glomerular and non-glomerular regions (even those collected from the same tissue sections). Each cluster of identified type is relatively tight without overlap between clusters, indicating that dimensionality reduction of Raman spectra using t-SNE can clearly discriminate between glomeruli constituting amyloid fibrils and normal glomerulus regions, and between AL and AA fibrils. We observed intra-group separation, especially in glomerular AA datapoints; however, the distance between the sub-groups is relatively small compared to the inter-group distances. As inter-group separation is significantly higher than intra-group separation, strong similarity among Raman spectra of the same types and regionality are observed from the t-SNE map. We attribute such clear separation between clusters, not only among different types but also between glomerular and non-glomerular regions, to the function of the glomerulus in the kidney. The glomerulus, a ball-shaped structure identified in Figure 4a, is responsible for filtering waste products and excess fluids from the blood [46]. As amyloidogenic proteins—serum amyloid A (AA) or immunoglobin light chain (AL)—form insoluble fibrils, they fail to pass through the filter; thus, most of these fibrils are deposited and accumulated in the glomeruli. Therefore, the amyloid protein deposits are predominantly found in the glomeruli [34,36]. This concentration of amyloid deposits in the glomeruli of AA and AL tissues is reflected in the Raman fingerprinting of the tissue, leading to clear separation in the t-SNE map.

**Figure 6:**
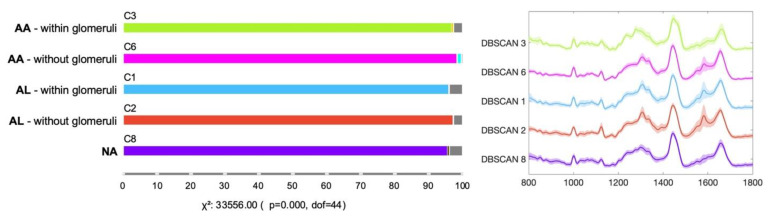
DBSCAN clustering results and representative Raman spectra of each cluster. (**Left**) Out of a total of 12 clusters, 5 dominant clusters were identified. AA glomerular and non-glomerular spectra are primarily grouped as Clusters 3 and 6, respectively. AL glomerular and non-glomerular spectra are primarily grouped as Clusters 1 and 2, respectively. NA tissue is primarily grouped as Cluster 8. The rest of the seven minor clusters are grouped accordingly. Unassigned spectra are marked as gray. (**Right**) Average spectra of the 5 dominant clusters with 1 standard deviation shaded.

Furthermore, DBSCAN results (Figure 6) obtained using the processed Raman tissue spectra between 800 and 1800 cm^−1^, show clustering results with distinctive separation among the types and glomeruli. DBSCAN analysis resulted in a total of 12 clusters, of which 5 major clusters represent 96.4% of the entire collection (8360 out of 8672 spectra) with parameters (number of neighbors as 2 within the radius of 1.09). The left panel of Figure 6 summarizes the arrangement of each cluster with respect to amyloid type and deposition site. 96.9% of glomerular AA (Cluster 3), 98.4% of non-glomerular AA (Cluster 6), 96% of glomerular AL (Cluster 1), and 97.2% of non-glomerular AL (Cluster 2) are identified as separate clusters. For the NA tissue, 95.6% of spectra are grouped as an individual cluster (Cluster 8). The remaining spectra are either unidentified or assigned to separate minor clusters. It is worth noting that these minor clusters do not have spectra pertaining to different amyloid types or deposition sites, demonstrating the robustness of the clustering analysis. The average spectra with one standard deviation shaded for the five major cluster groups are presented on the right panel of Figure 6. The spectral profiles demonstrate strong similarities to those of the actual spectra in Figure 4b, indicating that machine learning-based classification indeed enables us to characterize the types of amyloid fibrils and their deposition sites within the tissue.

In a previous study, we successfully utilized Raman spectroscopy to characterize crystal deposits in kidney biopsies [16], leading us to expand its application to the study of renal amyloid deposits. Spectroscopic techniques, including Raman spectroscopy, have demonstrated promise in detecting and identifying molecular changes in various kidney conditions [47,48]. With the aid of statistical and machine learning algorithms for analysis, these approaches can produce robust results [19,20,49]. Despite the limited sample size in this pilot study, Raman spectroscopy combined with appropriate analysis techniques was able to distinguish between different types of amyloids.

## 4. Conclusions

In this study, we characterized the Raman spectra of renal amyloid deposits within human tissues affected by systemic AL and AA amyloidosis. This label-free spectroscopic approach made it possible to obtain a biochemical fingerprint of unfixed, unstained specimens, providing intrinsic information on the content and structural profiles of ex vivo amyloid fibrils. Notably, Raman spectroscopy coupled with machine learning approaches exhibits multiple applications: one as a diagnostic tool that detects the presence of amyloid deposits and the other as a characterizing tool that can accurately distinguish AL and AA, two of the most common amyloid types in human kidney tissue. The collected Raman spectra of both glomerular and non-glomerular regions of all three tissue types, combined with t-SNE analysis, were able to identify subtle differences between samples and distinguish between AL, AA, and NA profiles, and even glomerular and non-glomerular regionality. Machine learning analysis equipped with DBSCAN distinguished AL and AA profiles based on their Raman spectra, suggesting the possibility of Raman spectroscopy as a tool for characterizing and subtyping amyloid.

Our label-free, machine learning-assisted spectroscopic analysis presents a new avenue for identifying amyloid within human tissue and promises an objective and reproducible diagnostic tool for systemic amyloidosis with renal involvement. While this study focused on fingerprinting features of AL and AA fibrils in frozen kidney sections, our methods could be extended to other systemic or hereditary amyloidoses in various organs.

## Data Availability

Not applicable.
